# Supplementary figures and images for: Hsp70 Gene Family in Sebastiscus marmoratus: The Genome-Wide Identification and Transcriptome Analysis under Thermal Stress
Source: Genes (Basel). 2023 Sep 9;14(9):1779. doi: 10.3390/genes14091779 (PMC10531354; doi:10.3390/genes14091779)

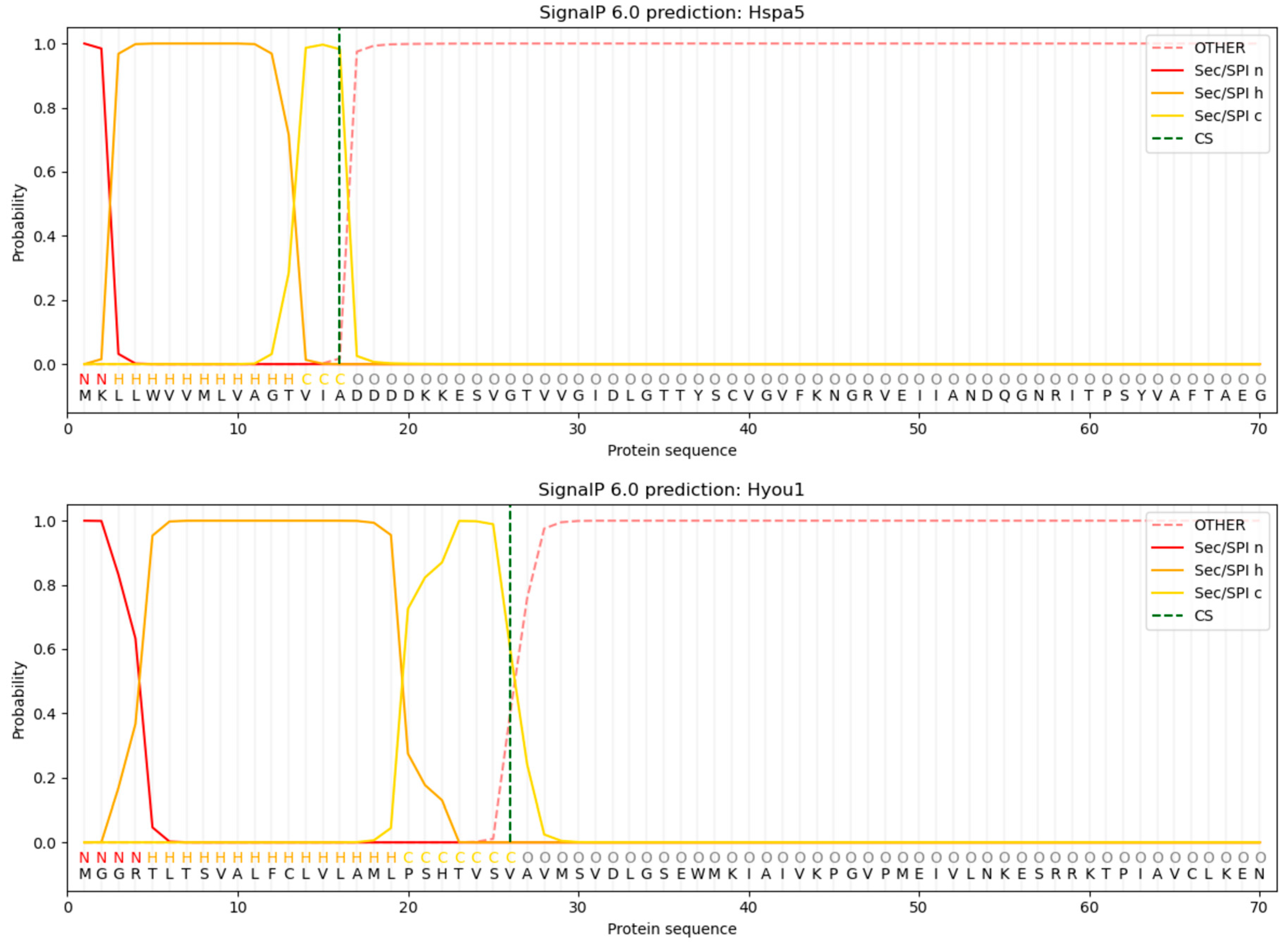

Supplement: Supplementary file 1 [file genes-14-01779-s001.zip › Figure S1.tif]
